# Self-assembling and pH-responsive protein nanoparticle as potential platform for targeted tumor therapy

**DOI:** 10.3389/fmolb.2023.1172100

**Published:** 2023-05-10

**Authors:** Zhikun Xu, Xiaozhan Zhang, Wang Dong, Huifang lv, Lijie Zuo, Lifei Zhu, Ruining Wang, Xia Ma

**Affiliations:** College of Veterinary Medicine, Henan University of Animal Husbandry and Economy, Zhengzhou, China

**Keywords:** trichosanthin, self-assembly, pH-Responsive, protein nanoparticle, tumor targeting

## Abstract

Frequent injections at high concentrations are often required for many therapeutic proteins due to their short *in vivo* half-life, which usually leads to unsatisfactory therapeutic outcomes, adverse side effects, high cost, and poor patient compliance. Herein we report a supramolecular strategy, self-assembling and pH regulated fusion protein to extend the *in vivo* half-life and tumor targeting ability of a therapeutically important protein trichosanthin (TCS). TCS was genetically fused to the N-terminus of a self-assembling protein, Sup35p prion domain (Sup35), to form a fusion protein of TCS-Sup35 that self-assembled into uniform spherical TCS-Sup35 nanoparticles (TCS-Sup35 NP) rather than classic nanofibrils. Importantly, due to the pH response ability, TCS-Sup35 NP well retained the bioactivity of TCS and possessed a 21.5-fold longer *in vivo* half-life than native TCS in a mouse model. As a result, in a tumor-bearing mouse model, TCS-Sup35 NP exhibited significantly improved tumor accumulation and antitumor activity without detectable systemic toxicity as compared with native TCS. These findings suggest that self-assembling and pH responding protein fusion may provide a new, simple, general, and effective solution to remarkably improve the pharmacological performance of therapeutic proteins with short circulation half-lives.

## 1 Introduction

Protein therapeutics are drawing more and more attention because they are biodegradable, metabolizable, highly active and specific (Leader et al., 2008; Walsh, 2018). Among them, ribosome-inactivating proteins (RIPs) that originate from different plant species have great potential as anticancer drugs due to their inhibitions of protein synthesis by inactive the ribosome, consequently, inducing apoptosis of eukaryotic cells (Bolognesi et al., 2016). In RIP family, trichosanthin (TCS) which is isolated from the root tuber of Chinese traditional medicine *trichosanthes kirilowii*, is gathering more and more attention and has been considered a potential cancer treatment drug (Lu et al., 2022). TCS has less cytotoxic effects than other RIPs such as ricin, abrin and luffaculin 1 (Sha et al., 2013), and more importantly, exhibits selective cytotoxicity against various cancers such as choriocarcinoma, breast cancer, leukemia, Lung cancer and colon carcinoma, etc (Zhu et al., 2021; Chen et al., 2022; Tan et al., 2022). However, the clinical use of TCS as a therapeutic is restrained due to their poor stability, immunogenicity, short circulation half-life, and especially side effect. For example, the molecular weight of TCS is only 27 kD, it can be easily cleared by kidney, the half-life in mice is only less than 3 h (Ko et al., 1991). As The short half-life makes necessary frequent administration (daily or thrice weekly) at high concentrations to maintain therapeutically effective blood drug levels, that will lead to adverse side effects such as myalgias, fevers and adverse neurological reactions. Besides, during clinical trials, occasional allergic reactions have been observed in patients receiving TCS treatment (Byers et al., 1990).

To solve these problems, common method is to enlarge protein size to prolong circulation time, then the administration dosage and side effects would be reduced. The attachment of poly (ethylene glycol) (PEG) to therapeutic proteins, called PEGylation, is one of the most frequently used methods to address these problems (Veronese, 2001; Pasut and Veronese, 2012). For instance, the half-life of TCS was extended to 65 h in human after PEGylation, thus lowering the administration dosage and side effects of TCS (Chen et al., 2017). However, PEGylation typically generates a heterogeneous product mixture of positional isomers which are difficult to separate and have reduced protein activity, due to its non-specific nature, PEGylation also affects the interaction between the protein drugs and the corresponding receptor, leading to poor tumor targeting and cellular uptake. Therefore, it is highly valuable to develop new methods to improve the *in vivo* half-life of therapeutic proteins and their tumoral targeting ability (Veronese, 2001; Gupta et al., 2019).

To increase the tumor targeting and cellular uptake of TCS, an alternative strategy is to develop self-assembled stimuli-responsive TCS nanoparticles that only accumulate in the specific tumor microenvironment, such as a relatively low pH of 5.4–7.0, which is widely used for pH-triggered theragnostic agents delivery (Bejarano et al., 2021; Chyuan et al., 2021). For example, many pH sensitive polymers or drugs were fused with proteins such as albumin to form nanoparticles (Deirram et al., 2019; Tian et al., 2019; Zhang et al., 2020), the size of those nanoparticle will enlarge at low pH tumor site, then they were accumulated at tumor site through the enhanced permeability and retention (EPR) effect, meanwhile, escape the filtration of the glomerulus, prolong the half-life in the body, reduce dosage and side effects.

Sup35p is a type of amyloid protein isolated from *Saccharomyces cerevisiae* (Glover et al., 1997; Serio et al., 2000). The N-terminal and middle region (containing the first 61 amino acid residues) of this protein, herein called Sup35, could self-assemble into β-sheet-rich amyloid nanofibrils with controlled sizes (King and Diaz-Avalos, 2004). These nanofibrils did not further form big aggregates and showed high solubility in many solutions (Men et al., 2009). Additionally, they were resistant to protein denaturation and proteases (Men et al., 2009; Men et al., 2010). Recently, Sup35 was fused with fluorescent proteins, enzymes, and other proteins to form hybrid protein nanofibrils for enhanced immunoassay (Schmuck et al., 2019), suggesting that Sup35 would serve as a general building block for constructing hybrid protein nanofibrils. However, in this study, we found that TCS-Sup35 could self-assemble into uniform core-shell spherical nanoparticles rather than classic nanofibrils. Furthermore, Sup35 fusion did no harm the secondary structure of TCS, so the TCS bioactivity *in vitro* was well retained. More importantly, TCS-Sup35 NP showed significantly improved pharmacokinetics, biodistribution and tumor targeting when compared with native TCS in mouse models. Consequently, TCS-Sup35 NP almost completely inhibited tumor growth without appreciable systemic toxicity in a mouse cancer model, whereas at the same dose, TCS was not so effective.

## 2 Materials and Methods

### 2.1 Construction, expression and purification of TCS-H6 and TCS-Sup35

The gene encoding TCS (GenBank: AY082349.2)and TCS-Sup35 was design in pET-25b (+) separately, and synthesized by Sangon company (Sangon Biotech, Shanghai), so the pET-TCS-H6 and pET- TCS-Sup35-H6 vector were obtained (H6 was 6× histidine tag for protein purification). After the DNA sequencing was verified, those recombinant plasmid pET-TCS-H6 and pET-TCS-Sup35 were transformed into *E. coli* Rosetta-gami B (DE3) plyss competent strain (Invitrogen) separately, and cultured in Terrific Broth (TB) medium containing 100 μg/mL ampicillin at 37°C. When the optical density at 600 nm (OD600) reached 0.5, the culture temperature was lowered to 25°C and then a final concentration of 0.5 mM IPTG was added for expression induction overnight.

Bacteria were collected and resuspended in lysis buffer (10 mM PB, 500 mM NaCl, 10 mM imidazole, pH = 7.4) and lysed by sonication. After centrifuged the matrix at 14,000 rpm for 20 min, polyethyleneimine (1% w/v) was added to the supernatant to precipitate nucleic acid. After centrifugation, collect supernatant and filtered with a 0.22 μm membrane, and then using a 5 mL HiTrap HP column (GE Healthcare) mounted on AKTA purifier to purify the protein with continuous gradient chromatography. Using 10 mM PB, 500 mM NaCl, 10 mM imidazole, pH = 7.4 as the equilibration buffer and 10 mM PB, 500 mM NaCl, 500 mM imidazole, pH = 7.5 as eluent. Peaks containing TCS or TCS-Sup35 were collected, exchanged to storing buffer (10 mM PB, 500 mM NaCl, pH = 7.5) via a desalting column. The protein samples were stored at −80°C.The solution buffer was changed to PBS (10 mM PB, 150 mM NaCl, pH = 7.5) before use.

The purification process was analyzed by SDS-PAGE. Protein concentration was determined by bicinchoninic acid (BCA) assay using a BCA kit (Beyotime Biotech) with directions.

### 2.2 Characterization of proteins

For dynamic light scattering (DLS), protein samples were diluted in pH of PBS, 10% Tween 20, or 10% SDS, followed by filtration with 0.22 μm pore size membranes. Protein number distribution was determined with a Zetasizer Nano-zs90 (Malvern) operating at a laser wavelength of 633 nm and a scattering angle of 90° at 25°C. Data were analyzed by Zetasizer software 6.32. For Circular dichroism (CD), Samples were diluted into a concentration of 0.2 mg/mL in H_2_O. CD spectra were recorded in the range from 190 nm to 260 nm on Pistar π-180 (Applied Photophysics Ltd.) instrument. For transmission electron microscopy (TEM), the morphologic and particle size examination of nanoparticles were performed by a Hitachi H-7650B transmission electron microscopy. The samples were diluted to 0.5 mg/mL, deposited onto carbon-coated copper grids for 2 min, stained with 2% (w/v) phosphotungstic acid solution, and air-dried at room temperature prior to imaging. For Critical micelle concentration (CMC), nile red was dissolved in PBS buffer to 1.25 μM. A series of dilutions of TCS-Sup35 from 8.3 μM to 0.03 μM were prepared for CMC measurement at 25 C and mixed with the nile red solution at a volume ratio of 1:1. The fluorescence intensity was determined at excitation/emission wavelengths of 550/630 nm with a SpectraMax M3 Microplate Reader (Molecular Devices).

### 2.3 *In vitro* anti-proliferation and biosafety study

Mouse breast cancer cells (4T1) and Mouse melanoma cells (B16) cells were cultured in RPMI-1640 medium with 15% fetal bovine serum (FBS) and 1% penicillin/streptomycin, and kept in a humidified, 5% CO_2_ atmosphere at 37 C. Human mammary epithelial cell (MCF-10A) and mouse fibroblast (L929) were cultured with DMEM medium with same condition described above. Cells were seeded in a 96 well plate (Corning) with a density around 5,000 cells/well and serial dilutions (20, 40, 80, 160, 320, 640, 1,280, and 2,560 ng/mL) of the samples in fresh medium were added. The wells filled with just media and media-treated cells served as the background and control, respectively, After incubating the plate for 72 h, cell viability was assessed using the MTT assay, as outlined in the Cell Proliferation Assay kit (Promega). Data fitting and IC_50_ calculation were then performed using Origin 9.0 software and the results were reported as the mean ± standard deviation.

### 2.4 Protein internalization study

First, for protein visualization, atto-488 NHS ester (Sigma) was conjugated with TCS and TCS-Sup35 proteins at a molar ratio 1:2 (protein/dye). Then conjugating efficiency was calculated using Nanodrop ND-1000. For confocal, after planting 4T1 cells on Mat-Teck culture dishes (Mat Teck Corp., Ashland, MA, United States) and incubating overnight, 100 ng/mL of protein was added to the cell culture and incubated for 12 h. The nucleus was then stained with Hoechst 33,342 (0.2 mg/mL, Molecular Probes) and the plasma membrane was stained with Cell MaskTM Deep Red (2.5 mg/mL, Molecular Probes). Once the incubation was completed in darkness for 5 min, the cells were washed with PBS for 3 times. The cells were then analyzed with a TCS-SP5 confocal laser scanning microscope (Leica Microsystems, Heidelberg, Germany) with a Plan Apo 639/1.4 (oil HC 9 PL APO l blue) objective.

### 2.5 Pharmacokinetics

Healthy female SPF BALB/c nude mice with an average body weight of about 20 g were randomly divided into 3 groups (6–8 weeks old, n = 3 per group) and intravenously injected with TCS and TCS-Sup35 at a dose of 1 mg TCS equivalent/kg body weight (BW), and an equivalent volume of PBS, respectively. At selected time points (0, 1, 5, 15, 30 min, 1, 3, 6, 24, 48, 72 and 96 h), blood samples (10 μL) were collected via tail vein, centrifuged at 4,000 *g* for 15 min after standing for 30 min at 4°C. The plasma was stored at −80 C for further use. TCS concentration in plasma was determined by ELISA assay under the instruction of ELISA kit (PBL Interferon Source). Pharmacokinetic parameters were analyzed by DAS 3.0 software and fitted with a two-compartment model.

### 2.6 Biodistribution

6–8 weeks old female BALB/c nude mice were inoculated subcutaneously with 5 × 10^6^ OVCAR-3 cells (0.2 mL) in the right dorsal area. When tumor size reached 100 mm^3^ (∼4 weeks), the mice were divided into 2 groups (3 mice in each group), and injected intravenously with TCS and TCS-Sup35 at a dosage of 1 mg TCS equivalent/kg BW, respectively. At 5 h after injection, mice were euthanized and major organ (tumor, heart, kidney, liver, spleen, lung, pancreas, and muscles) were collected, weighted and homogenized, and then resuspended in corresponding quantity of extraction buffer (10 mM PBS, 1 mM EDTA, 1% Triton X-100, 0.5% sodium deoxycholate, 1 mM PMSF, phosphatase inhibitor cocktail 2 and 3 and inhibitor cocktail (1:100 diluted)). The concentration of TCS in each sample was measured by ELLIA as described previously. Data were presented as percentage of total injected dose (%ID) per gram of tissue.

### 2.7 Anti-tumor efficacy

Human OVCAR-3 cells were cultured and implanted into female BALB/c nude mice as described above. When tumor size reached 30 mm^3^ (∼30 days), the animals were randomized to 3 groups (n = 6–8 per group), and acquired intravenous injections of TCS and TCS-Sup35 at a dosage of 50 μg equivalent TCS per mouse, and PBS at an equivalent volume once every 4 days until the mice of control group (PBS group) were sacrificed. Tumor volumes were measured via digital calipers and calculated with the formula: volume = length ×width^2^ × 0.5. The mice were weighed on the days of tumor measurements and would be killed if their tumor volume was larger than 500 mm^3^ or the body weight loss was greater than 15%. Statistical analyses were performed by Origin 9.0 software.

### 2.8 *In vivo* toxicity

To study the *in vivo* toxicity of TCS-Sup35, histomorphology, clinical biochemistry and hematology were examined after the treatment. After treated with PBS, TCS and TCS-Sup35 NP for 28 days, the mice were sacrificed and organs including tumor, heart, kidney and liver were collected, fixed with 4% neutral paraformaldehyde, and embedded in paraffin for histological examination. Tissue sections with a 5 μm thickness mounted onto glass slides were stained with hematoxylin-eosin (H&E) for morphology observation according to standard procedures. The images of all sections were captured with a Nikon Eclipse 90i microscope.

On day 28, a Hematology Analyzer (SYSMEX) was used to measure hematological parameters such as white blood cells (WBC), red blood cells (RBC), platelets (PLT), and the concentration of hemoglobin (HGB), and an Automatic Biochemical Analyzer (HITACHI) was employed to analyze serum lactate dehydrogenase (LDH), creatine kinase isoenzymes (CK-MB), aspartate aminotransferase (AST), alanine aminotransferase (ALT), creatinine (CRE) and blood urea nitrogen (BUN) in the collected blood sample.

The Laboratory Animal Facility at the Henan University of Animal Husbandry and Economy is accredited by AAALAC (Association for Assessment and Accreditation of Laboratory Animal Care International), and all animal protocols used in this study are approved by the Institutional Animal Care and Use Committee (IACUC).

## 3 Results and discussion

### 3.1 Protein NP purification and characterization

**FIGURE 1:**
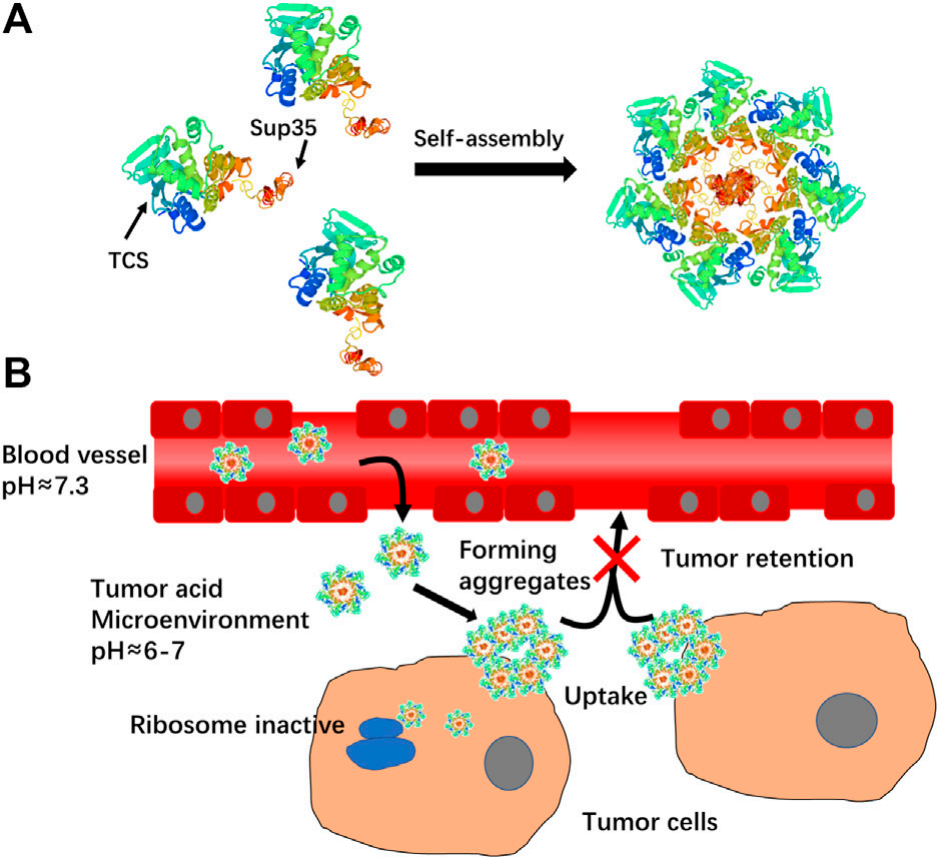
**(A)**Schematic illustration of self-assembly of TCS-Sup35 into nanoparticles. **(B)** Mechanism schematic illustration of TCS-Sup35 NP targeting to tumor site.

**FIGURE 2:**
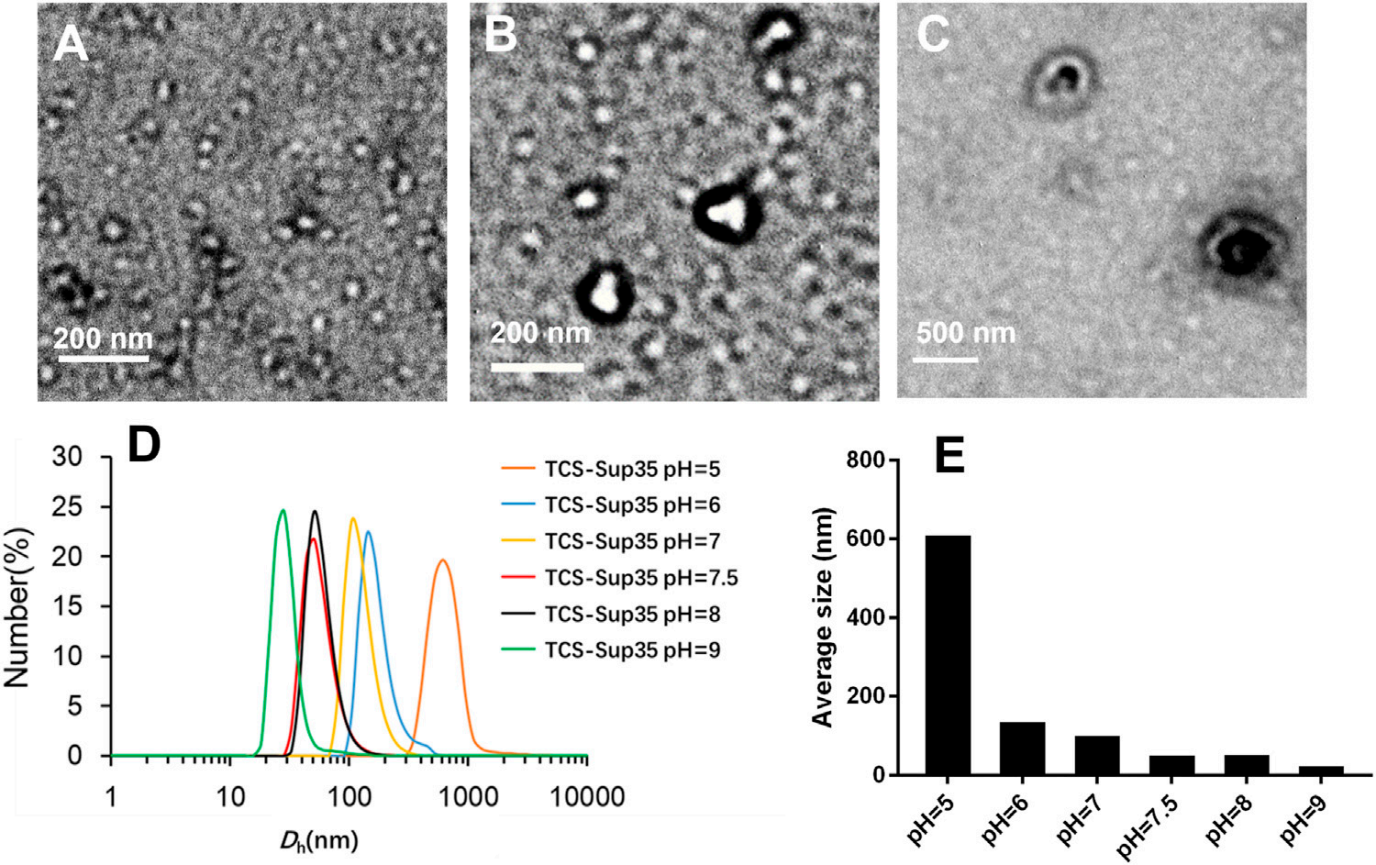
Physicochemical characterization of TCS-Sup35 NP. **(A)** TEM result of TCS-Sup35 NP in pH = 7.5 buffer. **(B)** TEM result of TCS-Sup35 NP in pH = 6 buffer. **(C)** TEM result of TCS-Sup35 NP in pH = 5 buffer. **(D)** DLS results of TCS-Sup35 NP in different pH buffers. **(E)** Average size of TCS-Sup35 NP in different buffers.

TCS-Sup35 and TCS were successfully expressed in *E. coli* after isopropyl β-D-1-thiogalactopyranoside (IPTG) induction, followed by purification with immobilized metal affinity chromatography. Sodium dodecyl sulfate polyacrylamide gel electrophoresis (SDS-PAGE) showed that TCS-Sup35 and TCS had expected molecular weights around 34 and 27 kDa, respectively (Supplementary Figure S1), which were well consistent with their theoretical values. Dynamic light scattering (DLS) showed that in pH = 7.5 buffer, TCS-Sup35 had a hydrodynamic diameter (*D*_h_) of around 55 nm, which was much larger than that of TCS (7 nm) (Figures 2D,E), suggesting self-assembly of TCS-Sup35 into nanoparticles (Figure 1A). Interestingly, when buffer pH decreased, the size of TCS-Sup35 was increased correspondingly, in pH = 5 buffer, the size of TCS-Sup35 reached 600 nm (Figure 2D). Transmission electron microscopy (TEM) further revealed uniform core-shell spherical nanoparticles with different sizes in different buffers (Figures 2A–C). This proved that TCS-Sup35 could self-assemble into protein nanoparticles with a larger size, and the size of this nanoparticle was pH responded. We further found that TCS-Sup35 NP dissociated into unimers after the addition of surfactants such as polyethylene glycol sorbitan monolaurate (tween 20) and sodium dodecyl sulfate (SDS) into the solution, as indicated by the size decrease from 55 nm to 5.6 and 4.8 nm, respectively (Supplementary Figure S3A). This result indicated that hydrophobic interaction and ionic bonding were two of the driving forces for self-assembly of TCS-Sup35 into spherical nanoparticles. In this condition, when the buffer pH decreased, the concentration of hydrogen ions increased, and the forces mentioned above will disrupt, leading to the disintegration of nanostructures and hydrophobic groups are exposed, resulting in the formation of protein aggregates. The critical micelle concentration (CMC) of TCS-Sup35 was measured to be as low as 0.55 μM (Supplementary Figure S3B). Moreover, circular dichroism (CD) showed that the secondary structure of TCS-Sup35 NP was α-helix instead of β-sheet, similar to that of TCS (Supplementary Figure S2). Surprisingly, this self-assembly behavior was different from those previously reported in which Sup35 fusion proteins self-assembled into β-sheet-rich amyloid nanofibrils (King and Diaz-Avalos, 2004; Schmuck et al., 2019). This result suggested that Sup35 fusion did not change the secondary structure of TCS but altered the self-assembly behavior of Sup35 from β-sheet-rich amyloid nanofibrils to α-helix-rich nanospheres. Taken together, these results implied that the Sup35 and TCS blocks of TCS-Sup35 formed the core and shell of the nanospheres, respectively (Figure 1A).

### 3.2 Anti-proliferative activity and biosafety

**FIGURE 3:**
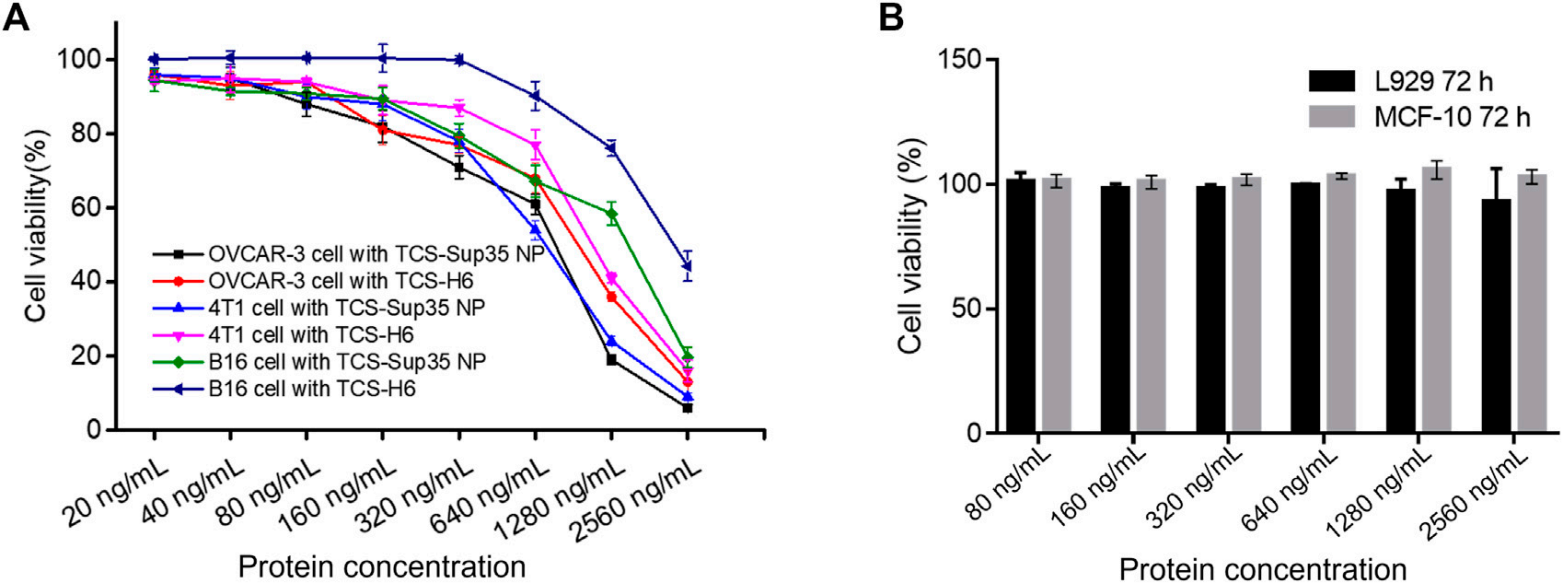
*In vitro* anti-proliferation of TCS-Sup35 NP and TCS-H6. **(A)** Cytotoxicity of TCS-H6 and TCS-Sup35 to OVCAR-3, 4T1 and B16 cells. **(B)** Cytotoxicity of TCS-Sup35 NP to L929 and MCF-10A cells. Data are shown as mean ± standard deviation (n = 3).

**FIGURE 4:**
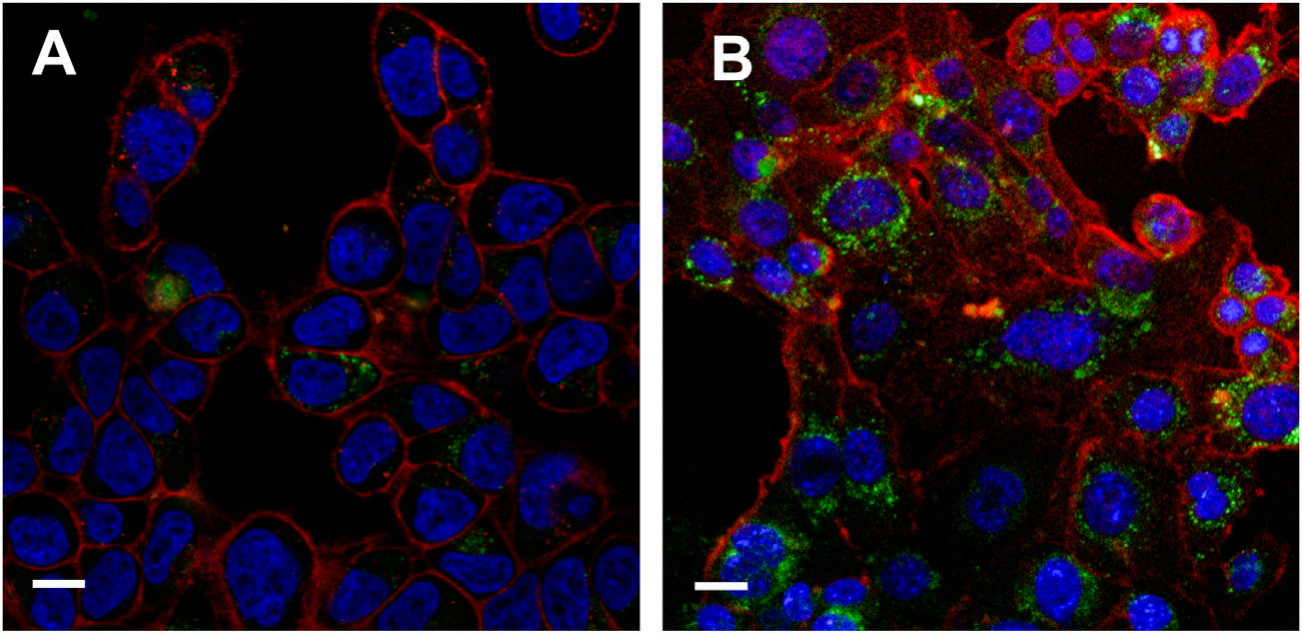
Internalization results of TCS-Sup 35 NP and TCS-H6 on 4T1 cells **(A)** Internalization result of TCS-H6 at 12 h. **(B)** Internalization result of TCS-Sup 35 NP at 12 h. The red color represents cytoplasm, blue color represents nuclear, green color represents proteins stained with FITC. The White bar indicate 15 μm.

Next, we measured the anti-proliferative activity of TCS-Sup35 NP by using OVCAR-3, 4T1 and B16 cells that are highly sensitive to TCS. From Figure 3A, we can find that at higher concentration group, the growth of OVCAR-3, 4T1 and B16 cells was significantly suppressed, the half maximal inhibitory concentration (IC_50_) of TCS-Sup35 NP and TCS toward OVCAR-3 were 604 and 947, respectively, and that in 4T1 cells were 615 and 1,130 ng/mL, respectively, indicating that the antiproliferative activity of TCS-Sup35 NP was nearly 1.7 times higher than native TCS. Compared to OVCAR-3 and 4T1 cells, B16 cells were less sensitive to TCS, the IC_50_ of TCS-Sup35 NP and TCS was 1,092 and 2,364 ng/mL, TCS-Sup35 NP still hold stronger tumor cell cytotoxicity than TCS. This may due to that after the formation of nanoparticle, the internalization ability was increased because more receptor was present on the surface of nanoparticle, thus, more TCS was transported inside tumor cells and the inhibition of tumor cells was also enhanced. This hypothesis was supported by confocal results. From Figures 4A,B, we can find that compare to TCS, the internalization efficacy of TCS-Sup 35 NP was significantly improved, which means more TCS-Sup35 NPs were transported inside 4T1 cells than TCS alone (Figure 4D), indicating that forming nanoparticle could enhance the permeability of TCS, and the Anti-proliferative activity was increased as well. On the other hand, TCS-Sup35 NP and TCS did not affect the viability of normal cells such as human mammary epithelial cells (MCF-10A) and mouse fibroblast cells (L929) (Figure 3B), indicating that Sup35 fusion did not cause any cytotoxicity to normal cells.

### 3.3 Pharmacokinetics and biodistribution

**FIGURE 5:**
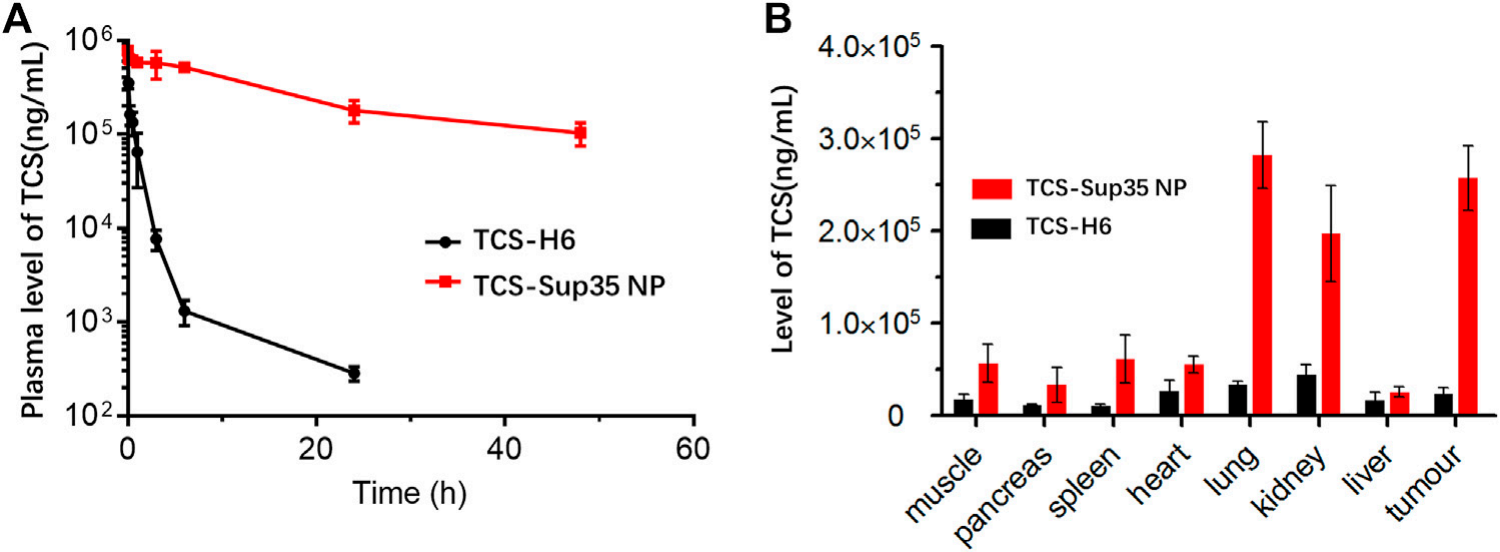
*In vivo* pharmacokinetics and biodistribution of TCS-Sup 35 NP and TCS-H6. **(A)** Plasma TCS concentration of TCS and TCS-Sup35 NP as a function of time post administration. **(B)** Biodistribution of TCS-Sup35 NP and TCS-H6 5 h after administration. Data are shown as mean ± standard deviation. (n = 3, *p < 0.05, **p < 0.01, significant difference between TCS-Sup35 NP and TCS).

The size of TCS-Sup35 NP (55 nm in diameter) was 5.5-fold larger than the cutoff size of kidney filtration (around 10 nm); in contrast, TCS was smaller in size (7 nm) than the cutoff size of renal clearance. These data suggested that TCS-Sup35 NP would enhance the *in vivo* half-life of TCS by reducing renal clearance due to its enlarged size. To verify this hypothesis, we investigated the pharmacokinetics of TCS-Sup35 NP in a mouse model (Figure 5A). After a single intravenous injection, the plasma concentration of TCS rapidly decreased; in contrast, TCS-Sup35 NP showed a much slow decrease in plasma level. These data indicated that TCS-Sup35 NP had a much slower renal clearance than TCS, as expected. The data were further fitted with a two-compartment model to obtain pharmacokinetic parameters (Supplementary Table S1). The terminal half-life (t_1/2β_ = 17.9 h) of TCS-Sup35 NP was 21.5-fold longer than that (0.83 h) of TCS. Especially, after 24 h, the TCS plasma level of TCS-Sup35 NP (180,241 ng/mL) was 632 times higher than that of TCS (285 ng/mL). Moreover, the area under the curve (AUC) increased by 51 times from 0.32 × 106 ng/mL/L*h for TCS to 16.6 × 106 ng/mL/L*h for TCS-Sup35 NP, indicating the significantly improved systemic exposure of TCS-Sup35 NP relative to TCS. All these results showed that Sup35 fusion could substantially enhance the pharmacokinetic performance of TCS in a mouse model.

As the pH of the tumor micro-environment was around 5–7, we assumed that TCS-Sup 35 NP could target to tumor site through EPR effect and retained there because TCS-Sup 35 NP formed larger size particle at lower pH (Figure 1B). To prove that, we further examined the biodistribution of TCS-Sup35 NP in major organs and tissues in an ovarian tumor-bearing mouse model at 5 h post a single intravenous injection (Figure 5B). TCS-Sup35 NP had higher levels than TCS in heart, kidney, liver, lung, spleen, pancreas, muscle and tumor. Particularly, the tumor concentration of TCS-Sup35 NP (257,197 ng/g tissue) was 11.3-fold higher than that of TCS (22,846 ng/g tissue), indicating the high tumor targeting efficiency of TCS-Sup35 NP presumably owing to their high serum level and the enhanced permeation and retention (EPR) effect. Additionally, TCS-Sup35 NP also showed a much higher level in lung than TCS, probably due to the presence of the intricate blood capillary distribution and huge amount of alveolar macrophages in lung that tend to take up large particles. Interestingly, the kidney concentration of TCS-Sup35 NP was 4.5-fold higher than that of TCS, suggesting that TCS-Sup35 NP could be cleared from the body through kidney filtration likely due to their dissociation into unimers with a smaller size at low concentrations than the cutoff size of kidney filtration. Collectively, these results indicated that TCS-Sup35 NP could markedly improve the biodistribution of TCS in a tumor-bearing mouse model.

### 3.4 Anti-tumor efficacy

**FIGURE 6:**
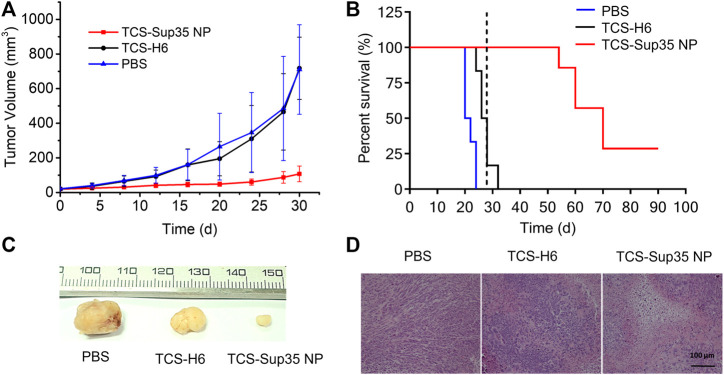
*In vivo* anti-tumor efficacy. **(A)** Inhibition of tumor growth, in which the arrows denote the injection time points. **(B)** Cumulative survival of mice, the vertical line indicates the end of treatment. Data are shown as mean ± standard deviation. (n = 6–7, **p < 0.01, significant difference between TCS-Sup35 NP and TCS, TCS-Sup35 and PBS). **(C)** Representative images of tumors in each group. **(D)** H&E staining of tumor tissues after the treatments.

Encouraged by the significantly improved pharmacokinetics and biodistribution of TCS-Sup35 NP as compared to TCS, we further investigated the anti-tumor efficacy of TCS-Sup35 NP in an ovarian cancer mouse model (Figure 6). After tumors reached an average size of 30 mm^3^ at ∼30 d post inoculation of cancer cells, mice were randomized into three groups, and treated with PBS, TCS, and TCS-Sup35 NP, respectively, at the same dose of 50 μg TCS equivalent per mouse every 4 days until the tumor size of control groups reached 500 mm^3^. Tumor growth was slightly inhibited in TCS treatment group as compared to PBS treatment group (Figure 6A and Supplementary Figure S4). In contrast, tumor growth was significantly suppressed in TCS-Sup35 NP group. On day 28 post administration, the tumor size in TCS-Sup35 NP treatment group (87.5 mm^3^) was 5.3 and 5.5 folds smaller than those in TCS treatment group (465 mm^3^) and PBS treatment group (485 mm^3^), respectively (Figure 6C). The outperformance of TCS-Sup35 NP in inhibiting tumor growth over TCS led to a substantial increase of animal survival (Figure 6B). All of mice treated with PBS and TCS suffered from death at 24 and 32 d post injection, respectively. In contrast, 75% mice treated with TCS-Sup35 NP were subjected to death at 70 d post administration, and the left 25% were still alive with small sizes of tumors (48 mm^3^ on average) that did not grow any more. Hematoxylin-Eosin (H&E) staining further confirmed the anti-tumor efficacy of TCS-Sup35 NP (Figure 6D). Not surprisingly, severe damage to tumor cells was observed in TCS-Sup35 NP treatment group as compared to PBS treatment group; in contrast, slight tumor cell damage was detected in TCS treatment group. These results showed that Sup35 fusion could remarkably improve the anti-tumor efficacy of TCS in a tumor-bearing mouse model due to its enhanced tumor targeting and cell penetration ability.

### 3.5 *In vivo* toxicity

The biosafety of TCS-Sup35 NP treatment was further studied. The body weight of mice in all treatment groups remained steady during the treatments. H&E staining indicated that TCS-Sup35 NP and TCS treatments did not induce any significant histological change in major organs such as heart, liver and kidney at 28 d post administration as compared with PBS treatment (Supplementary Figure S5). The biochemistry parameters of major organs such as liver, heart, and kidney from each group did not change significantly (Supplementary Figure S6). Additionally, hematological markers such as white blood cells, red blood cells, hemoglobin and platelets in each group did not alter too much (Supplementary Figure S7). Collectively, these results showed that TCS-Sup35 NP treatment did not induce detectable systemic toxicity.

## 4 Conclusion

In summary, we have presented a novel strategy to extend the *in vivo* half-life of an important therapeutic protein, TCS, by generating a pH-regulating and self-assembling protein nanoparticle with significant tumor targeting and inhibiting ability. Our results demonstrate that genetic fusion of TCS to Sup35 produces self-assembled TCS-Sup35 NP with uniform spherical morphology, instead of the β-sheet-rich amyloid nanofibrils that were always observed for Sup35 alone and its fusion proteins before. Unlike TCS-HAS or PEGylated-TCS (Ren et al., 2022; Chen et al., 2017), TCS-Sup35 NP can be produced in *E. coli* with high efficiency, indicating low production cost. Moreover, the bioactivity of TCS-Sup35 NP is much higher than TCS alone, and its internalization ability was vastly enhanced. More interestingly, Sup35 fusion not only substantially prolongs the *in vivo* half-life of TCS but also markedly improves the biodistribution of TCS in a mouse model. This is because the size of the nanoparticle increases when pH decreases, allowing the nanoparticle to accumulate at the tumor site and enhance the tumor targeting ability and the delivery efficacy of TCS to the tumor site. Consequently, Sup35 fusion remarkably enhances the anti-tumor activity of TCS in a tumor-bearing mouse model without detectable side effects. These findings suggest that Sup35 fusion can offer a supramolecular property to therapeutic proteins, such as interferon alpha/beta/gamma/lambda, erythropoietin, granulocyte colony-stimulating factor, epidermal growth factor, insulin-like growth factor, and glucagon-like peptide-1, to tremendously extend their *in vivo* half-lives, improve their biodistribution, and thus enhance their therapeutic efficacy. We believe that other self-assembling proteins (Bai et al., 2016; Zhang and Orner, 2011), such as virus capsid proteins and ferritin, would also be applicable to improve the *in vivo* half-life of therapeutic proteins. Therefore, self-assembling protein fusion may provide a new, simple, and effective technology platform to improve the pharmacological properties of therapeutic proteins with short circulation half-lives.

## Data Availability

The datasets presented in this study can be found in online repositories. The names of the repository/repositories and accession number(s) can be found in the article/Supplementary Material.

## References

[B1] Bai Y. Luo Q. Liu J. (2016). Protein self-assembly via supramolecular strategies. Chem. Soc. Rev. 45, 2756–2767. 10.1039/c6cs00004e 27080059

[B2] Bejarano L. Jordao M. J. C. Joyce J. A. (2021). Therapeutic targeting of the tumor microenvironment. Cancer Discov. 11, 933–959. 10.1158/2159-8290.CD-20-1808 33811125

[B3] Bolognesi A. Bortolotti M. Maiello S. Battelli M. G. Polito L. (2016). Ribosome-inactivating proteins from plants: A historical overview. Molecules 21, 1627. 10.3390/molecules21121627 27898041 PMC6273060

[B4] Byers V. S. Levin A. S. Waites L. A. Starrett B. A. Mayer R. A. Clegg J. A. (1990). A phase I/II study of trichosanthin treatment of HIV disease. AIDS 4, 1189–1196. 10.1097/00002030-199012000-00002 2128454

[B5] Chen G. Xiong W. Gu Z. Gao Y. Hou J. Long L. (2022). Mannosylated engineered trichosanthin-legumain protein vaccine hydrogel for breast cancer immunotherapy. Int. J. Biol. Macromol. 223, 1485–1494. 10.1016/j.ijbiomac.2022.11.045 36395942

[B6] Chen Y. Zhang M. Jin H. Tang Y. Wu A. Xu Q. (2017). Prodrug-like, PEGylated protein toxin trichosanthin for reversal of chemoresistance. Mol. Pharm. 14, 1429–1438. 10.1021/acs.molpharmaceut.6b00987 28195491

[B7] Chyuan I. T. Chu C. L. Hsu P. N. (2021). Targeting the tumor microenvironment for improving therapeutic effectiveness in cancer immunotherapy: Focusing on immune checkpoint inhibitors and combination therapies. Cancers (Basel) 13, 1188. 10.3390/cancers13061188 33801815 PMC7998672

[B8] Deirram N. Zhang C. Kermaniyan S. S. Johnston A. P. R. Such G. K. (2019). pH-responsive polymer nanoparticles for drug delivery. Macromol. Rapid Commun. 40, e1800917. 10.1002/marc.201800917 30835923

[B9] Glover J. R. Kowal A. S. Schirmer E. C. Patino M. M. Liu J. J. Lindquist S. (1997). Self-seeded fibers formed by Sup35, the protein determinant of [PSI+], a heritable prion-like factor of *S. cerevisiae* . Cell 89, 811–819. 10.1016/s0092-8674(00)80264-0 9182769

[B10] Gupta V. Bhavanasi S. Quadir M. Singh K. Ghosh G. Vasamreddy K. (2019). Protein PEGylation for cancer therapy: Bench to bedside. J. Cell Commun. Signal 13, 319–330. 10.1007/s12079-018-0492-0 30499020 PMC6732144

[B11] King C. Y. Diaz-Avalos R. (2004). Protein-only transmission of three yeast prion strains. Nature 428, 319–323. 10.1038/nature02391 15029195

[B12] Ko W. H. Wong C. C. Yeung H. W. Yung M. H. Shaw P. C. Tam S. C. (1991). Increasing the plasma half-life of trichosanthin by coupling to dextran. Biochem. Pharmacol. 42, 1721–1728. 10.1016/0006-2952(91)90508-3 1718284

[B13] Leader B. Baca Q. J. Golan D. E. (2008). Protein therapeutics: A summary and pharmacological classification. Nat. Rev. Drug Discov. 7, 21–39. 10.1038/nrd2399 18097458

[B14] Lu J. Q. Wong K. B. Shaw P. C. (2022). A sixty-year research and development of trichosanthin, a ribosome-inactivating protein. Toxins (Basel) 14, 178. 10.3390/toxins14030178 35324675 PMC8950148

[B15] Men D. Guo Y. C. Zhang Z. P. Wei H. P. Zhou Y. F. Cui Z. Q. (2009). Seeding-induced self-assembling protein nanowires dramatically increase the sensitivity of immunoassays. Nano Lett. 9, 2246–2250. 10.1021/nl9003464 19402649

[B16] Men D. Zhang Z. P. Guo Y. C. Zhu D. H. Bi L. J. Deng J. Y. (2010). An auto-biotinylated bifunctional protein nanowire for ultra-sensitive molecular biosensing. Biosens. Bioelectron. 26, 1137–1141. 10.1016/j.bios.2010.07.103 20970983

[B17] Pasut G. Veronese F. M. (2012). State of the art in PEGylation: The great versatility achieved after forty years of research. J. Control Release 161, 461–472. 10.1016/j.jconrel.2011.10.037 22094104

[B18] Ren Z. Zhao J. Cao X. Wang F. (2022). Tandem fusion of albumin-binding domains promoted soluble expression and stability of recombinant trichosanthin *in vitro* and *in vivo* . Protein Expr. Purif. 200, 106147. 10.1016/j.pep.2022.106147 35917982

[B19] Schmuck B. Gudmundsson M. Hard T. Sandgren M. (2019). Coupled chemistry kinetics demonstrate the utility of functionalized Sup35 amyloid nanofibrils in biocatalytic cascades. J. Biol. Chem. 294, 14966–14977. 10.1074/jbc.RA119.008455 31416835 PMC6791322

[B20] Serio T. R. Cashikar A. G. Kowal A. S. Sawicki G. J. Moslehi J. J. Serpell L. (2000). Nucleated conformational conversion and the replication of conformational information by a prion determinant. Science 289, 1317–1321. 10.1126/science.289.5483.1317 10958771

[B21] Sha O. Niu J. Ng T. B. Cho E. Y. Fu X. Jiang W. (2013). Anti-tumor action of trichosanthin, a type 1 ribosome-inactivating protein, employed in traditional Chinese medicine: A mini review. Cancer Chemother. Pharmacol. 71, 1387–1393. 10.1007/s00280-013-2096-y 23377374 PMC3668121

[B22] Tan Y. Xiang J. Huang Z. Wang L. Huang Y. (2022). Trichosanthin inhibits cell growth and metastasis by promoting pyroptosis in non-small cell lung cancer. J. Thorac. Dis. 14, 1193–1202. 10.21037/jtd-22-282 35572907 PMC9096284

[B23] Tian Q. Li Y. Jiang S. An L. Lin J. Wu H. (2019). Tumor pH-responsive albumin/polyaniline assemblies for amplified photoacoustic imaging and augmented photothermal therapy. Small 15, e1902926. 10.1002/smll.201902926 31448572

[B24] Veronese F. M. (2001). Peptide and protein PEGylation: A review of problems and solutions. Biomaterials 22, 405–417. 10.1016/s0142-9612(00)00193-9 11214751

[B25] Walsh G. (2018). Biopharmaceutical benchmarks 2018. Nat. Biotechnol. 36, 1136–1145. 10.1038/nbt.4305 30520869

[B26] Zhang B. Wan S. Peng X. Zhao M. Li S. Pu Y. (2020). Human serum albumin-based doxorubicin prodrug nanoparticles with tumor pH-responsive aggregation-enhanced retention and reduced cardiotoxicity. J. Mater Chem. B 8, 3939–3948. 10.1039/d0tb00327a 32236239

[B27] Zhang Y. Orner B. P. (2011). Self-assembly in the ferritin nano-cage protein superfamily. Int. J. Mol. Sci. 12, 5406–5421. 10.3390/ijms12085406 21954367 PMC3179174

[B28] Zhu C. Zhang C. Cui X. Wu J. Cui Z. Shen X. (2021). Trichosanthin inhibits cervical cancer by regulating oxidative stress-induced apoptosis. Bioengineered 12, 2779–2790. 10.1080/21655979.2021.1930335 34233587 PMC8806483

