# Corrigendum: Self-assembling and pH-Responsive protein nanoparticle as potential platform for targeted tumor therapy

**DOI:** 10.3389/fmolb.2023.1232803

**Published:** 2023-06-22

**Authors:** Zhikun Xu, Xiaozhan Zhang, Wang Dong, Huifang lv, Lijie Zuo, Lifei Zhu, Ruining Wang, Xia Ma

**Affiliations:** College of Veterinary Medicine, Henan University of Animal Husbandry and Economy, Zhengzhou, China

**Keywords:** trichosanthin, self-assembly, pH-Responsive, protein nanoparticle, tumor targeting

In the published article, there was an error in the **Funding** statement. The funding information was missing. The correct Funding statement appears below.

“This work supported by Henan Province key research and promotion special project: 212102110374. Key scientific research project of Henan Province:21B230004, 20A230005, Starting Research Fund from the Henan University of Animal Husbandry and Economy: 2019HNUAHEDF004, National Natural Science Foundation of China (NSFC): 31902274 and National Natural Science Foundation of China: 32072906.”

The authors apologize for this error and state that this does not change the scientific conclusions of the article in any way. The original article has been updated.

