# Supplementary material for: Self-assembling and pH-responsive protein nanoparticle as potential platform for targeted tumor therapy
Source: Front Mol Biosci. 2023 May 10;10:1172100. doi: 10.3389/fmolb.2023.1172100 (PMC10206137; doi:10.3389/fmolb.2023.1172100)
Supplement: Supplementary file 1 [file DataSheet1.DOCX]

Supplementary Material

Self-assembling and pH-Responsive Protein Nanoparticle as Potential Platform for Targeted Tumor Therapy

**Zhikun Xu^1^*, Xiaozhan Zhang^1^, Wang Dong^1^, Huifang lv^1^, Lijie Zuo^1^**

College of Veterinary Medicine, Henan University of Animal Husbandry and Economy, Zhengzhou, 4500167, P. R. China.

*** Correspondence:**Zhikun Xu
xuzhikun@hnuahe.edu.cn

## 1. Supplementary Figures

##
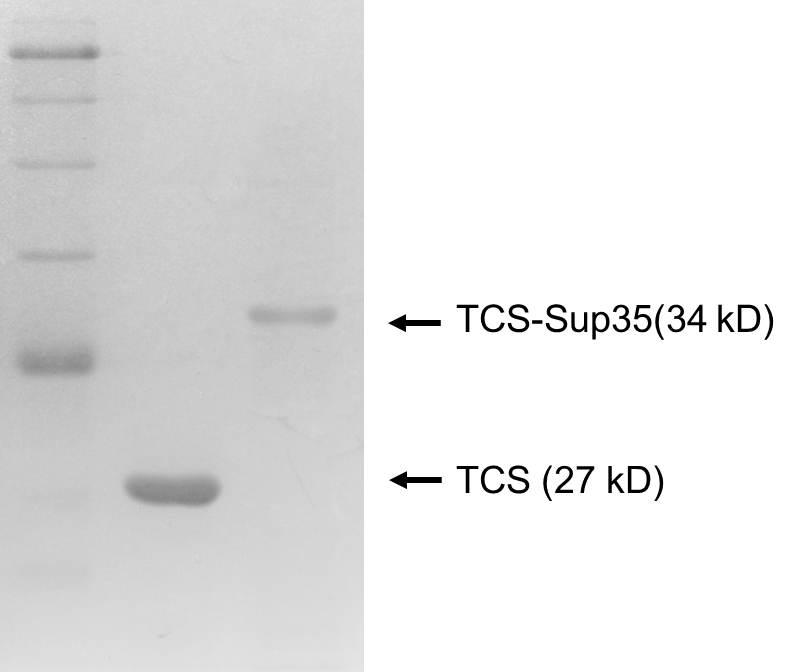


## Supplementary Figure 1. SDS-PAGE analysis of TCS and TCS-Sup35.


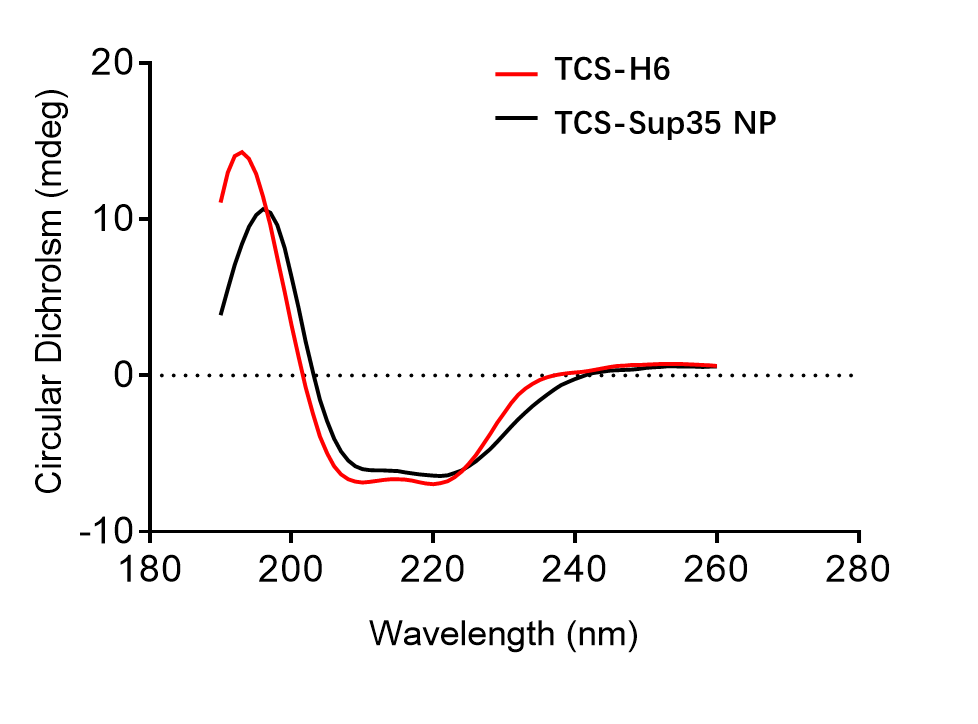


**Supplementary Figure 2.** CD spectra of TCS-Sup35 NP and TCS.


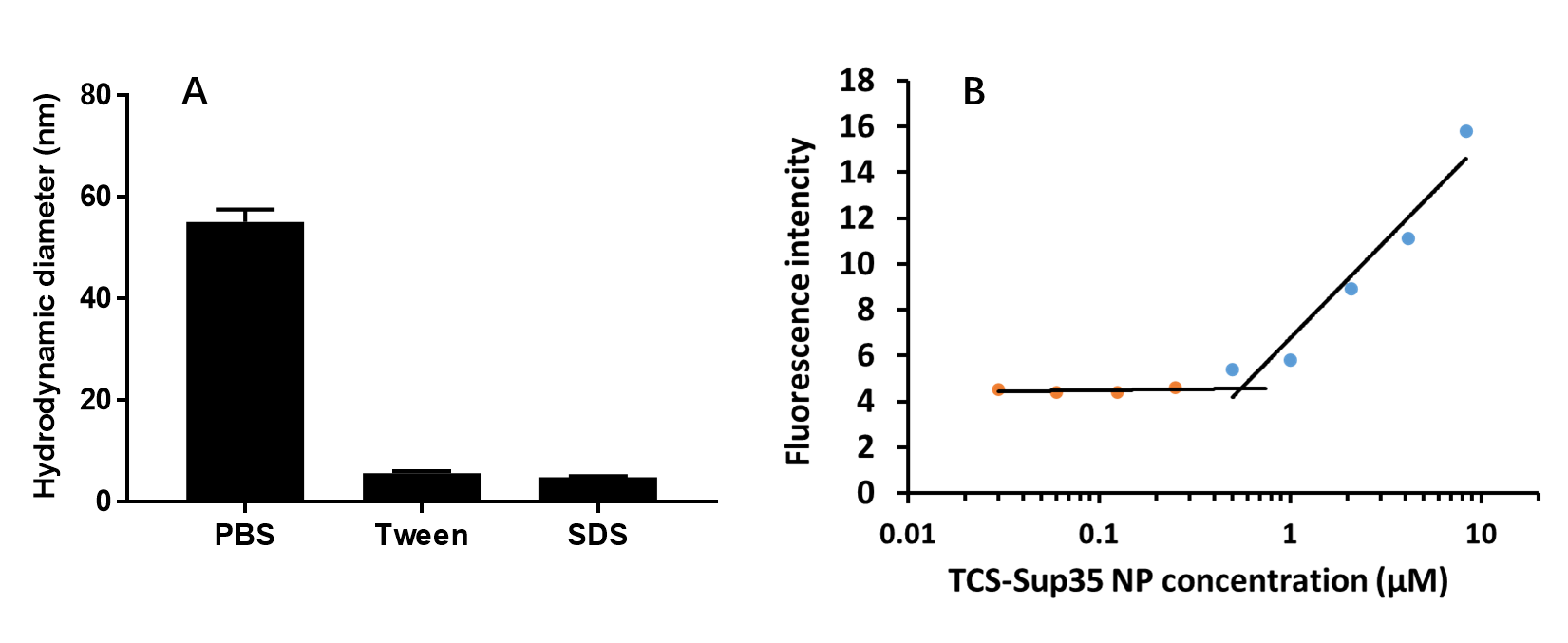


**Supplementary Figure 3.** A: DLS analysis of TCS-Sup35 NP in PBS, PBS containing 10% Tween 20 and 10% SDS. B: Measurement of CMC of TCS-Sup35 NP in PBS.


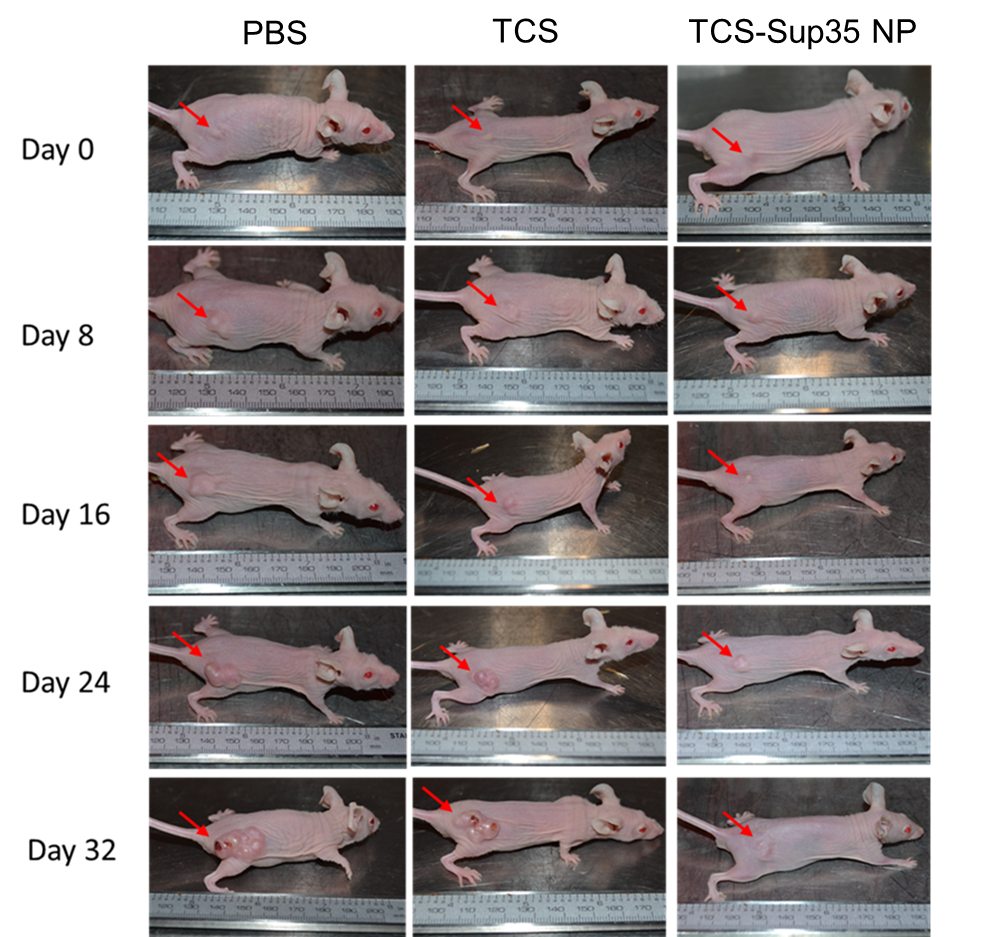


**Supplementary Figure 4.** Representative images for the tumor growth post administration. Arrows indicate the positions of tumor.


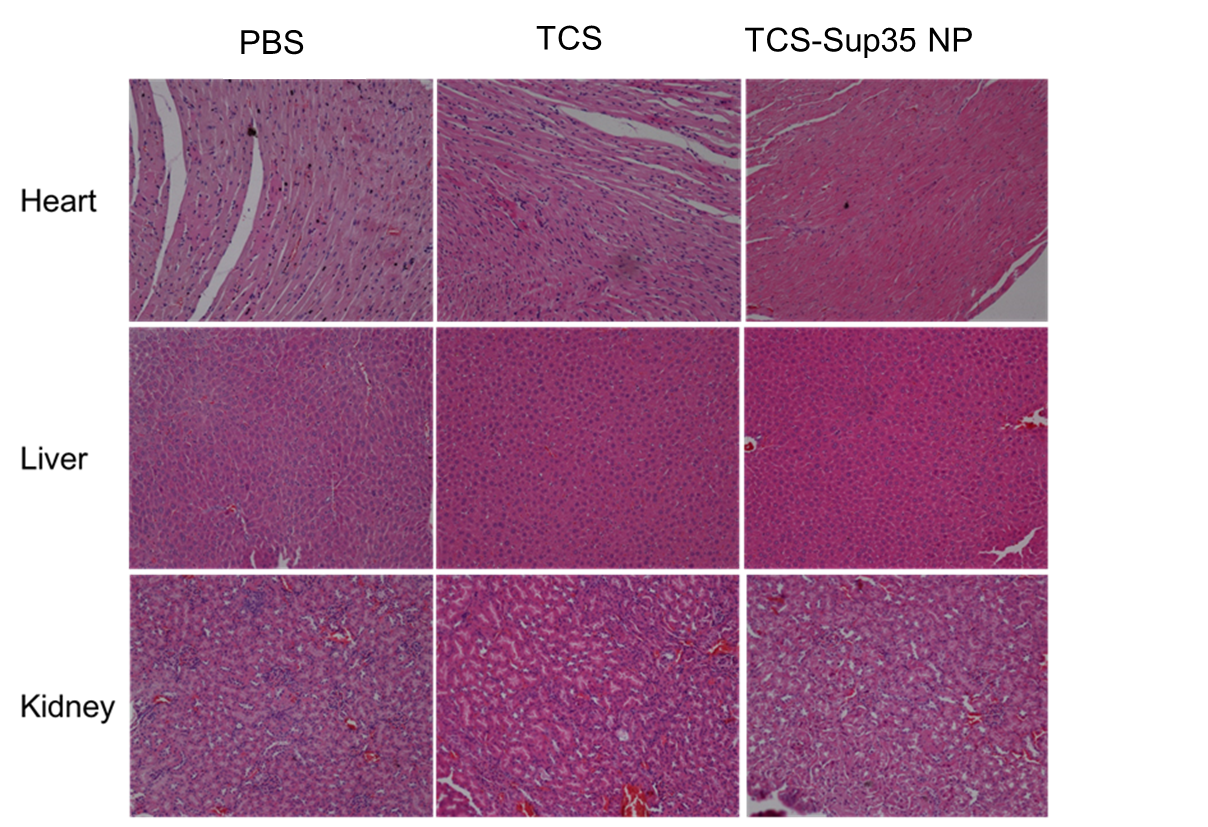


**Supplementary Figure 5.** Representative H&E staining of different organs after the treatments.


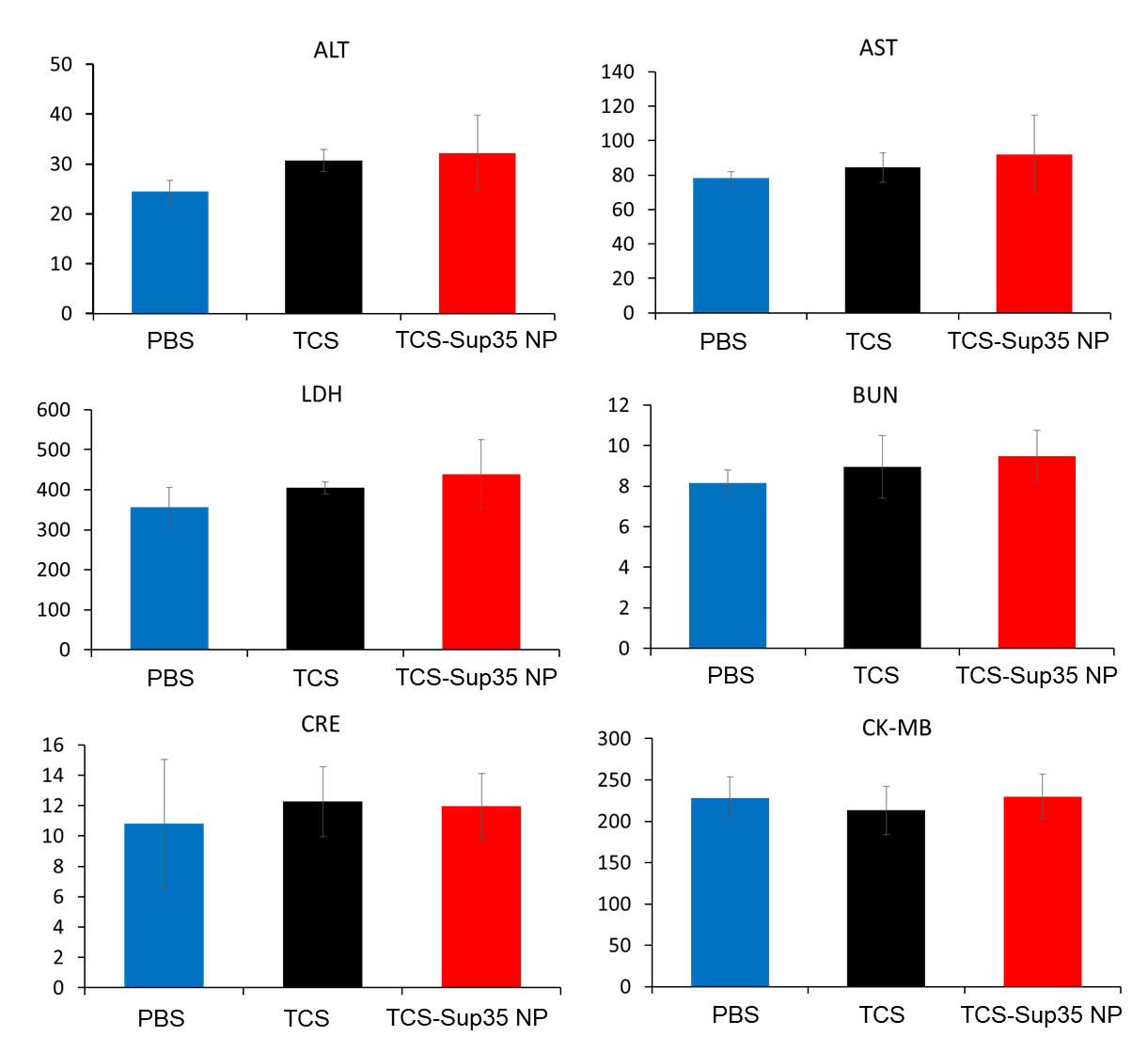


**Supplementary Figure 6.** Clinical biochemistry parameters for the mice at 28 days post administration. Liver function markers: ALT(alanine aminotransferase); AST(aspartate aminotransferase). Heart function markers: LDH(lactate dehydrogenase); CK-MB(creatine kinase isoenzymes). Kidney function markers: CRE(creatinine); BUN(blood urea nitrogen). Data are shown as mean ± standard deviation (n = 6-7).


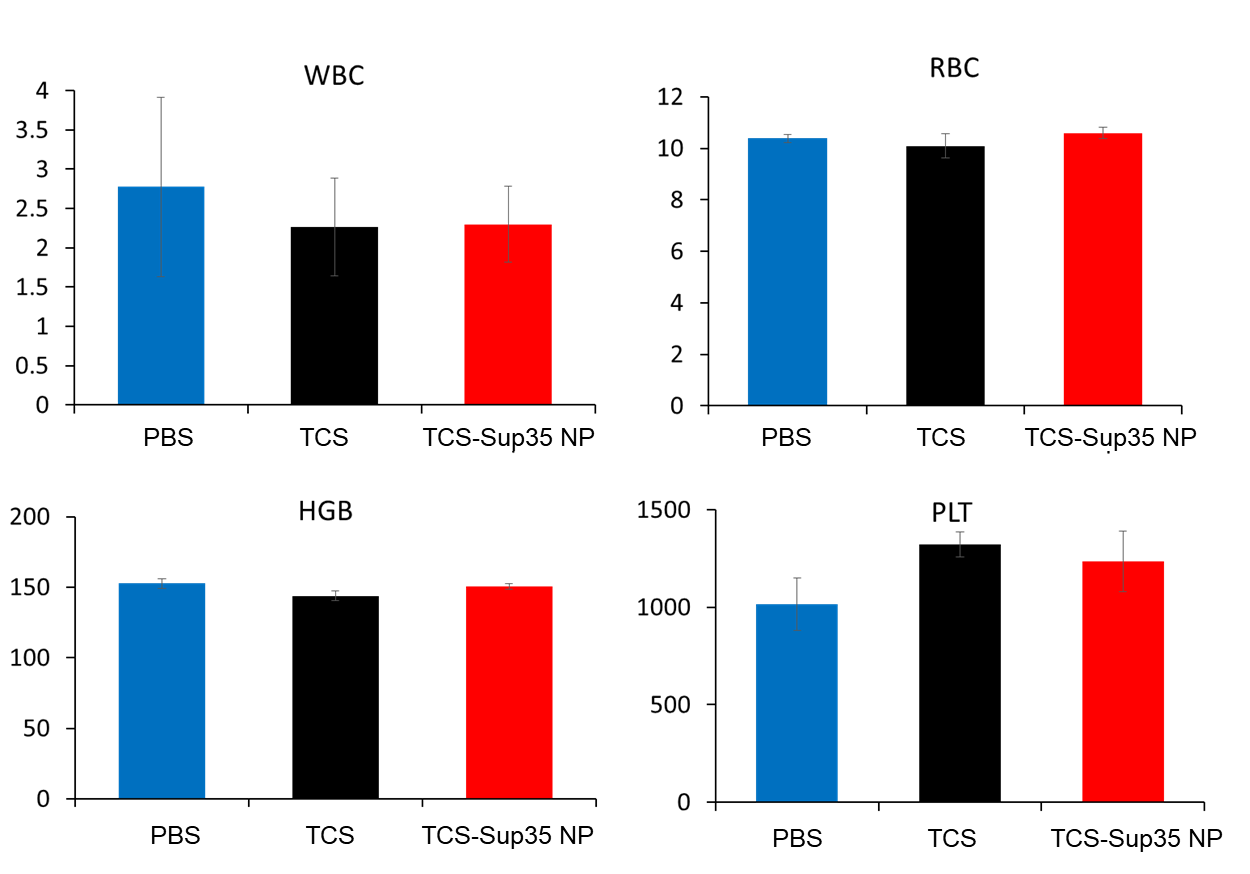


**Supplementary Figure 7.** Hematological parameters for the mice at 28 days post administration. WBC, white blood cells; RBC, red blood cells; PLT, platelets; HGB, hemoglobin. Data are shown as mean ± standard deviation (n = 6-7).

**2. Supplementary table**

**Supplementary Table 1.** Pharmacokinetic parameters of TCS and TCS-Sup35 NP.

| **Parameters** | **TCS** | **TCS-Sup35 NP** |
| --- | --- | --- |
| Distribution half-life t1/2α (h) | 0.06 ± 0.02 | 0.18 ± 0.09 |
| Terminal half-life t1/2β (h) | 0.83 ± 0.25 | 17.9 ± 1.8 |
| Central compartment volume of distribution  V1 (L/kg) | 29.8 ± 2.3 | 30.6 ± 2.3 |
| Peripheral compartment volume of distribution  V2 (L/kg) | 53.0 ± 4.1 | 1.8± 0.4 |
| Central clearance CL1 (L/h/kg) | 83.6± 7.4 | 1.26± 0.97 |
| Peripheral clearance CL2 (L/h/kg) | 173.3 ± 42.3 | 6.35 ± 1.46 |
| Area under curve AUC(0-∞) (10^6^ pg/mL/L*h) | 0.32 ± 0.05 | 16.6 ± 1.3 |
| Elimination rate constant K10 (1/h) | 2.81 ± 0.85 | 0.041± 0.00 |
| Central to peripheral rate constant K12 (1/h) | 5.82 ± 2.12 | 0.208± 0.03 |
| Peripheral to central rate constant K21 (1/h) | 3.26 ± 1.32 | 3.577± 2.02 |
